# Potential activity of *Ferula macrecolea* essential oil for treating *Giardia lamblia* infection through modulating electrolytes and suppressing NF-κB p65 pathway

**DOI:** 10.3389/fphar.2025.1542425

**Published:** 2025-02-18

**Authors:** Iraj Salimikia, Seyed Ehsan Yaghoubi, Amal Khudair Khalaf, Leila Masoori, Javad Ghasemian Yadegari, Hossein Mahmoudvand

**Affiliations:** ^1^ Department of Pharmacognosy, Lorestan University of Medical Sciences, Khorramabad, Iran; ^2^ Department of Microbiology, College of Medicine, University of Thi-qar, Thi-qar, Iraq; ^3^ Razi Herbal Medicines Research Center, Lorestan University of Medical Sciences, Khorramabad, Iran; ^4^ Hepatitis Research Center, Lorestan University of Medical Sciences, Khorramabad, Iran

**Keywords:** herbal medicines, natural products, giardiasis, inflammation, diarrhea

## Abstract

**Background:**

The pharmacological treatment of *Giardia lamblia* infection involves the use of chemical agents, such as metronidazole (MNZ). However, these medications are associated with a range of adverse effects, and their effectiveness is not definitively established. In light of the previously discussed information and the recognized antimicrobial properties of *Ferula macrecolea*, this study aims to investigate both the *in vitro* and *in vivo* anti-giardial effects of *F. macrecolea* essential oil (FME) on *G. lamblia* infection.

**Methods:**

Gas chromatography-mass spectrometry (GC-MS) was utilized to analyze the chemical composition of the prepared FME. The MTT colorimetric assay was employed to assess FME’s *in vitro* anti-giardial and cytotoxic activities. FME’s *in vivo* effects were evaluated compared to MNZ in mice infected with *G. lamblia*. Additionally, the effects of FME therapy on serum electrolyte levels and the expression levels of inflammatory cytokines were assessed.

**Results:**

The primary components of FME were identified as terpinolene (78.72%), n-nonanal (4.47%), and linalool (4.35%). FME significantly reduced the viability and growth rate of *G. lamblia* trophozoites (IC_50_ = 21.6 μg/mL) and cysts (IC_50_ = 27.6 μg/mL) in a dose-dependent manner compared to the control group (p < 0.001). The CC_50_ value for FME against normal intestinal cells was determined to be 207.4 μg/mL. *In vivo*, assays demonstrated that the administration of various doses of FME, particularly in combination with MNZ over 7 days, resulted in a statistically significant reduction in the mean number and viability of *Giardia* cysts, serum level electrolytes (sodium and potassium), and the expression levels of interleukin-1 (*IL-1*), tumor necrosis factor-alpha (*TNF-α*), nuclear factor κB p65 (*NF-κB p65*), and Toll-like receptor 4 (*TLR-4*) in mice with giardiasis (p < 0.001).

**Conclusion:**

This study’s results demonstrate the extract’s efficacy *in vitro* against *G. lamblia*, exhibiting minimal cytotoxicity towards normal cells. Furthermore, the extract was shown to manage giardiasis in murine models by modulating electrolyte levels and inflammatory responses via suppressing the NF-κB p65/TLR pathways. However, further research is necessary to clarify the specific efficacy and mechanisms of action of the extract in combating *G. lamblia* infection.

## 1 Introduction

*Giardia lamblia* is a flagellated protozoan that resides in the human small intestine and has been reported worldwide (Adam, 2001). The primary mode of human infection is through the ingestion of contaminated food and water, as well as through direct fecal-oral transmission. The durability of the parasite’s cysts against chlorination processes in water increases its potential for transmission via aquatic sources. Although infection can occur at any age, it is notably more common among children (Laishram et al., 2012). Current estimates suggest that there are approximately 280 million cases of human infection each year. *G. lamblia*, the causative agent of giardiasis, is widespread in both temperate and tropical regions and is linked to clinical symptoms such as diarrhea (steatorrhea), particularly in pediatric populations, as well as malabsorption, abdominal cramps, and weight loss (Hooshyar et al., 2019).

The pharmacological treatment of this disease entails the use of chemical agents, including metronidazole (MNZ) and furazolidone (Watkins and Eckmann, 2014). However, these medications are linked to a variety of adverse effects, and their effectiveness is not definitively established. Moreover, certain agents have been reported to exhibit carcinogenic and mutagenic characteristics, particularly in vulnerable populations such as women and children, and are contraindicated during pregnancy (Watkins and Eckmann, 2014; Vivancos et al., 2018). Additionally, there are documented cases of parasitic resistance to these pharmacological agents (Watkins and Eckmann, 2014; Vivancos et al., 2018). As a result, there is an increasing interest in research focused on identifying alternative compounds that demonstrate fewer or no adverse effects. Recent research work has underscored the therapeutic potential of certain herbs and their derivatives, including *Zataria* spp., *Eucalyptus* spp., and *Allium* spp., in the management of *Giardia* infections (Alnomasy et al., 2021). However, the wider utilization of herbal remedies for giardiasis is currently hindered by inconclusive research findings that frequently lack adequate empirical validation.

The *Ferula* genus, comprising over 150 distinct species, is recognized as one of the most extensively utilized groups of plants in traditional medicine globally (Yaqoob and Nawchoo, 2016). Among these, *Ferula macrecolea* Boiss stands out due to its diverse therapeutic properties, which include analgesic, anti-inflammatory, antihypertensive, antibacterial, anti-parasitic, antiviral, antifungal, and insecticidal effects (Yaqoob and Nawchoo, 2016; Salehi et al., 2019). This plant plays a significant role in the treatment of cardiovascular and gastrointestinal disorders within both traditional and contemporary medical practices (Yaqoob and Nawchoo, 2016; Salehi et al., 2019). Recent studies have identified terpenoid compounds, such as terpinolene, α-pinene, and myrcene, as the primary constituents of the essential oils derived from various *Ferula* species (Boghrati and Iranshahi, 2019). The chemical composition of essential oils is influenced by several factors, including the specific plant part utilized, the timing of the harvest, the geographical location of the harvest, and the method of extraction employed (Sahebkar and Iranshahi, 2011; Asili et al., 2009). In light of the previously discussed information and the recognized antimicrobial properties of the *F. macrecolea*, this study seeks to investigate both the *in vitro* and *in vivo* anti-giardial effects of the *F. macrecolea* essential oil (FME) on *G. lamblia* infection.

## 2 Material and methods

### 2.1 Plant collection

In the present study, the aerial portions of the plant were collected from the western regions of Islamabad, located in Kermanshah province, during April 2021. Following confirmation and identification by a botanist, an herbarium specimen (No. 1400.2276) was prepared and subsequently deposited at the herbarium of the Razi Herbal Medicines Research Center at Lorestan University of Medical Sciences in Khorramabad, Iran. The plant materials were then dried, ground into a powder, and stored in opaque containers to ensure preservation.

### 2.2 Preparing of the essential oil

The essential oil was isolated utilizing the hydro-distillation method employing a Clevenger apparatus. The extraction process lasted for a duration of 4 h. Following the extraction, the essential oil was separated from the aqueous phase and subsequently dried using sodium sulfate. The extracted essential oil was stored at refrigeration temperatures in clean containers wrapped in aluminum foil until the chemical and antigiardial assays were conducted (Mahmoudvand et al., 2017).

### 2.3 Gas chromatography-mass spectrometry (GC-MS)

GC-MS was employed to analyze the chemical composition of the prepared essential oil. The analysis utilized an HP6890-Packard-Hewlett gas chromatograph, which was fitted with a 30-meter-long column with a diameter of 25 mm and a stationary phase thickness of 0.25 μm, specifically of the 5MS-HP type. The temperature program for the column commenced at an initial temperature of 50°C, maintained for 5 min, followed by a temperature ramp to 250°C at a rate of 5°C per minute, with a final hold at 250°C for 20 min. Helium was used as the carrier gas at a flow rate of 1 mL/min. The mass spectrometer employed was an Agilent Model 5,975, operating with an ionization voltage of 70 eV, utilizing electron impact ionization, and an ionization source temperature set at 220°C. The identification of the essential oil compounds was conducted by comparing their retention indices and mass spectra against the WILEY 09 and NIST 11 mass spectral databases (Adams, 2004; NIST, 2014).

### 2.4 Cells culture

Normal human intestinal epithelial cells (NCM460) were procured from the Pasteur Institute located in Tehran, Iran. These cells were cultured in Dulbecco’s Modified Eagle’s Medium (DMEM, Sigma-Aldrich, Germany), which was supplemented with 10% fetal bovine serum, and were maintained at a temperature of 37°C in an atmosphere containing 5% CO_2_.

### 2.5 Parasite

To isolate *G. lamblia* cysts, stool samples were obtained from patients diagnosed with giardiasis who were referred to healthcare facilities in Khorramabad, Iran. Positive samples were confirmed through direct examination and the formalin-ether concentration technique. Subsequently, the sucrose gradient method (0.85 M) was utilized to concentrate the *Giardia* cysts. The cysts were then diluted with distilled water (DW) in a 12:1 ratio, and the resulting solution was filtered. An additional 5 mL of DW was added to the sediment, and the upper phase was carefully combined with 3 mL of sucrose solution. This mixture was centrifuged at 700 g for 10 min at 4°C. The cysts were subsequently extracted from the middle layer using a Pasteur pipette, washed with normal saline, and stored at 4°C until further analysis. The concentration of the cysts was adjusted to 1 × 10^5^ cysts/mL using a hemocytometer (Ghasemian Yadegari et al., 2022).

### 2.6 *G. lamblia* trophozoites collection

The excystation of *Giardia* cysts to yield trophozoite forms was conducted in accordance with established protocols (Bingham and Meyer, 1979). In brief, an induction solution comprising aqueous hydrochloric acid was introduced to the cyst suspension at a ratio of 1:9 and incubated at 37°C for 120 min. Following this incubation, the suspension underwent centrifugation at 600 rpm for 10 min, after which the supernatant was removed, and the sediment was resuspended in a medium consisting of filter-sterilized TYI-S-33 culture, supplemented with bovine bile, 20% heat-inactivated fetal calf serum, streptomycin (500 μg/mL), and penicillin (500 IU/mL). The resulting mixture was subsequently incubated at 37°C in a slant culture.

### 2.7 *In vitro* antigiardial effects of FME

An initial volume of 100 µL containing trophozoite and cysts at a concentration of 1 × 10^5^ cells/mL was separately dispensed into each well of a 96-well plate. Subsequently, various concentrations of the FME and MNZ were separately added to the wells containing the cells, followed by incubation at 24°C for a duration of 48 h. After the supernatant was removed from the wells, 20 µL of MTT solution (0.5 mg/mL in PBS) was introduced into each well and incubated for 4 h in a 5% CO_2_ atmosphere at 37°C. Following the addition of 100 µL of dimethyl sulfoxide, the absorbance of each well was measured at 570 nm using an ELISA plate reader. The 50% inhibitory concentrations (IC_50_) was calculated using Probit analysis in SPSS software (Version 26.0) (Masoori et al., 2024).

### 2.8 Cytotoxic effects of FME

At first, 100 µL containing normal NCM460 cells at a concentration of 1 × 10^5^ cells/mL was separately dispensed into each well of a 96-well plate. Subsequently, various concentrations of the FME were separately added to the wells containing the cells, followed by incubation at 24°C for a duration of 48 h. After the supernatant was removed from the wells, 20 µL of MTT solution was introduced into each well and incubated for 4 h in a 5% CO_2_ atmosphere at 37°C. Following the addition of 100 µL of dimethyl sulfoxide, the absorbance of each well was measured at 590 nm using an ELISA plate reader. The 50% cytotoxic concentration (CC_50_) was calculated using Probit analysis in SPSS software (Version 26.0) (Mahmoudvand et al., 2016).

### 2.9 *In vivo* effects on giardiasis in mice

#### 2.9.1 Ethics

The study obtained ethical approval from the ethics committee of Lorestan University of Medical Sciences in Khorramabad, Iran, with the ethics identification number IR.LUMS.REC.1400.200.

#### 2.9.2 Animals

A cohort of sixty male BALB/C mice, aged 8–10 weeks and weighing between 25 and 30 g, was housed under optimal conditions, with continuous access to food and water.

#### 2.9.3 Study design

The mice were infected via oral administration of 0.2 mL of a cyst solution (2000 cysts). Subsequently, the animals were monitored through stool examination (SE), employing methods such as direct smear and the formalin-ether concentration technique, until the presence of *Giardia* cysts was confirmed in their feces (Masoori et al., 2024). After a period of 6 days, the mice were divided into six groups, each receiving one of the following treatments orally for 1 week including:

(i) Normal saline
(ii) MNZ at 15 mg/kg/day
(iii) FME at dose of 5 mg/kg/day
(iv) FME at dose of 10 mg/kg/day.
(v) FME at dose of 5 mg/kg/day + MNZ at 7.5 mg/kg/day
(vi) FME at dose of 10 mg/kg/day + MNZ at 7.5 mg/kg/day

#### 2.9.4 Antiparasitic effects on giardiasis in mice

On the eighth day post-treatment, stool examinations were conducted to assess the presence of *Giardia* cysts and to determine the reduction rate of these cysts. Additionally, the viability of the collected cysts was evaluated using the eosin exclusion assay, where pink cysts were classified as dead and colorless cysts as viable (Masoori et al., 2024).

### 2.10 Blood collection

In accordance with the established protocol, the experimental mice were subjected to deep euthanasia via intraperitoneal injection of a ketamine (100 mg/kg) + xylazine (10 mg/kg). Following the administration of deep anesthesia, an incision was made to access the abdominal cavity, from which blood samples were obtained directly from the cardiac region and were centrifuged, whereas the resultant serum was analyzed for biochemical analysis.

### 2.11 Evaluating the serum level electrolytes

The objective of this analysis was to quantify the serum level electrolytes of sodium (Na+) and potassium (K+) using diagnostic biochemical kits produced by Parsazmon, Iran.

### 2.12 Evaluating the expression level of proinflammatory cytokines

Total RNA was extracted utilizing Total RNA Extraction Kit, Favaorgen, Iran from the duodenal tissue of the tested mice. Subsequently, complementary DNA (cDNA) was generated employing the cDNA synthesis Kit, Yekta Tajhiz Azma, Iran. Quantitative real-time polymerase chain reaction (qRT-PCR) was performed using SYBR Green PCR master mix sourced from Yekta Tajhiz Azma, Iran. Table 1 presents the primers designed to target genes associated with inflammatory cytokines (Masoori et al., 2024). The protocol commenced with an initial denaturation step at 96°C for a duration of 8 min, followed by 40 cycles of amplification, and concluded with a final extension at 76°C for 4 min. Subsequently, the 2^−ΔΔCT^ method was employed for analysis, which was performed utilizing the Bio-Rad iQ5 Optical System Software, United States.

**TABLE 1:** The sequence of primer used in this study.

Primer	Sequence (5′–3′)
*TNF-α*	F: TGAACTTCGGGGTGATCGGT R: GGTGGTTTGTGAGTGTGAGGG
*IL-1β*	F: AACCTGCTGGTGTGTGACGTTC R: CAGCACGAGGCTTTTTTGTTGT
*NF-κB p65*	F: AGGCAAGGAATAATGCTGTCCTG R: ATCATTCTCTAGTGTCTGGTTGG
*TLR4*	F: AGCTTTGGTCAGTTGGCTCT R: CAGGATGACACCATTGAAGC
*β-actin*	F: GTGACGTTGACATCCGTAAAGA R: GCCGGACTCATCGTACTCC

### 2.13 Statistical analysis

After collecting the data and entering it into the SPSS 24 statistical software, appropriate measures of central tendency and dispersion were calculated. A one-way analysis of variance (ANOVA) were employed to analyze the data. If the results were significant, Tukey’s *post hoc* tests were conducted for paired comparisons. In cases where the data do not follow a normal distribution, alternative non-parametric tests were utilized. The significance level was set at p < 0.05.

## 3 Results

### 3.1 GC/MS analysis

The findings presented in Table 2 indicate that the GC/MS analysis identified a total of 18 compounds, accounting for 99.99% of the composition. The predominant components of the FME were identified as terpinolene (78.72%), n-nonanal (4.47%), and linalool (4.35%), respectively.

**TABLE 2:** Gas chromatography-mass spectrometry analysis of essential oil of *Ferula macrocolea*.

Constituent	RI	Percent (%)
α─pinene	936	0/11
p─cymene	1,010	0/23
β─phellandrene	1,028	1/31
Benzeneacetaldehyde	1,032	1/35
α─Thujene	1,035	3/92
Limonene	1,036	0/25
Methyl carvacrol	1,076	1/94
Terpinolene	1,094	78/72
Geijerene	1,098	0/58
n-Nonanal	1,102	4/47
α─campholenal	1,125	0/32
Linalool	1,139	4/35
Camphor	1,148	0/24
Thuj-3-en-lo-al	1,186	0/29
Myrtenal	1,196	1/22
Allo-ocimene	1,198	0/42
di-sec-butyl disulfide	1,212	0/17
Piperiton	1,252	0/1
Total		99/99

### 3.2 *In vitro* antigiardial effects against trophozoite and cyst of *G. lamblia*

**FIGURE 1:**
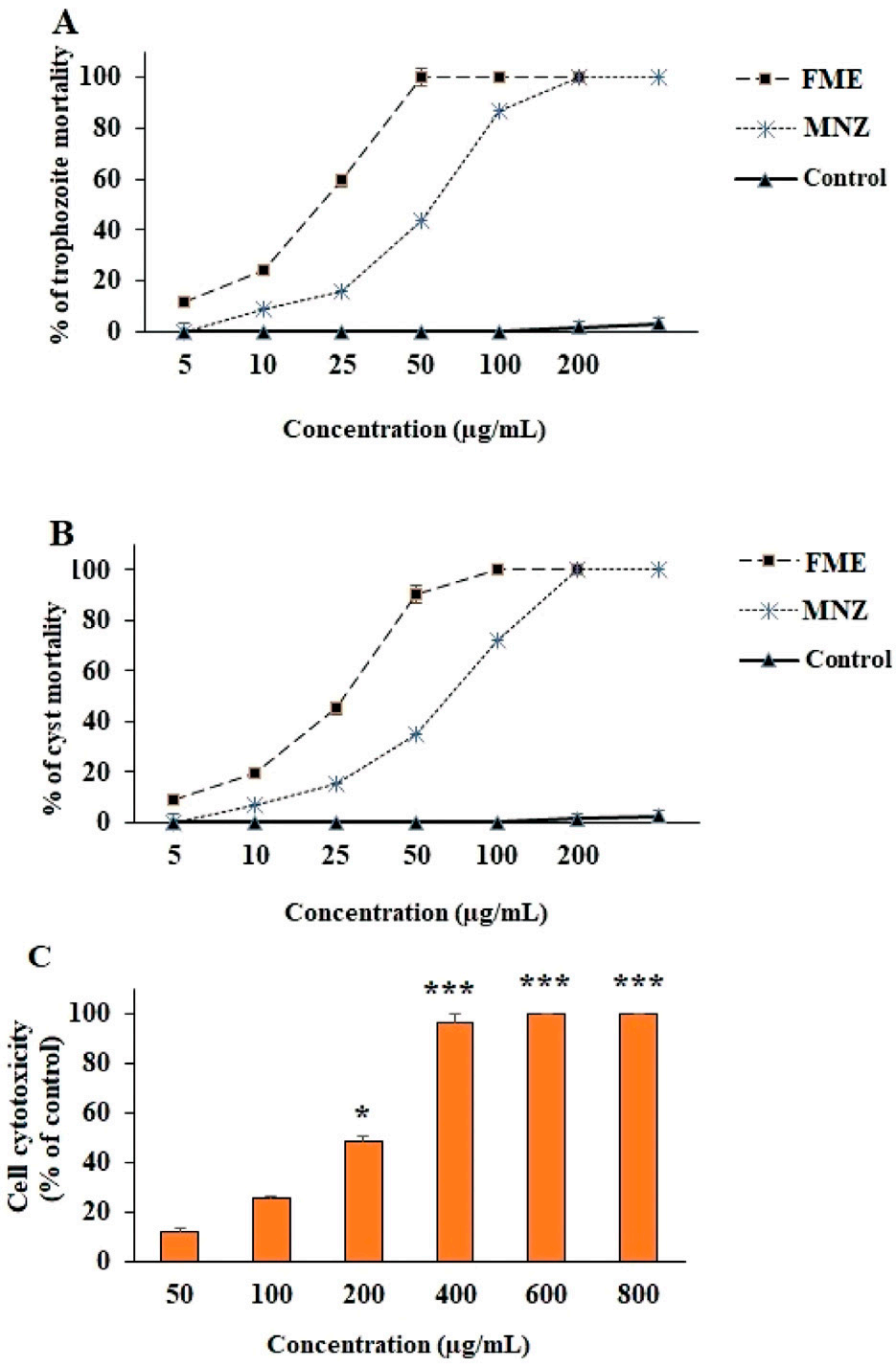
The effect of different concentrations of *Ferula macrecolea* essential oil (FME) on the trophozoites **(A)** and cysts **(B)** of *Giardia lamblia* in comparison to metronidazole (MNZ). The cytotoxicity of FME on normal human intestinal epithelial cells **(C)**. The results are presented as mean ± standard deviation (SD) with a sample size of n = 3.

The data illustrated in Figure 1 demonstrate that FME and MNZ, significantly (p < 0.001) diminished the viability and growth rate of *G. lamblia* trophozoites and cysts in a dose-dependent manner when compared to the control group. The IC_50_ values for FME and MNZ against *G. lamblia* trophozoites were determined to be 21.6 ± 2.43 and 28.7 ± 2.15 μg/mL, respectively. Furthermore, the IC_50_ values for FME and MNZ against *G. lamblia* cysts were determined to be 27.6 ± 1.51 and 35.6 ± 3.22 μg/mL, respectively.

### 3.3 Cytotoxicity effects of FME against normal intestinal cells

Figure 1C illustrates the cytotoxic effects of FME on both intestinal normal and cancerous cells, as assessed by the MTT assay. The findings indicate that the FME significantly decreased cell viability in a dose-dependent manner. The calculated CC_50_ values for FME against normal NCM460 cells were determined to be 207.4 μg/mL.

### 3.4 *In vivo* effects on giardiasis in mice

**FIGURE 2:**
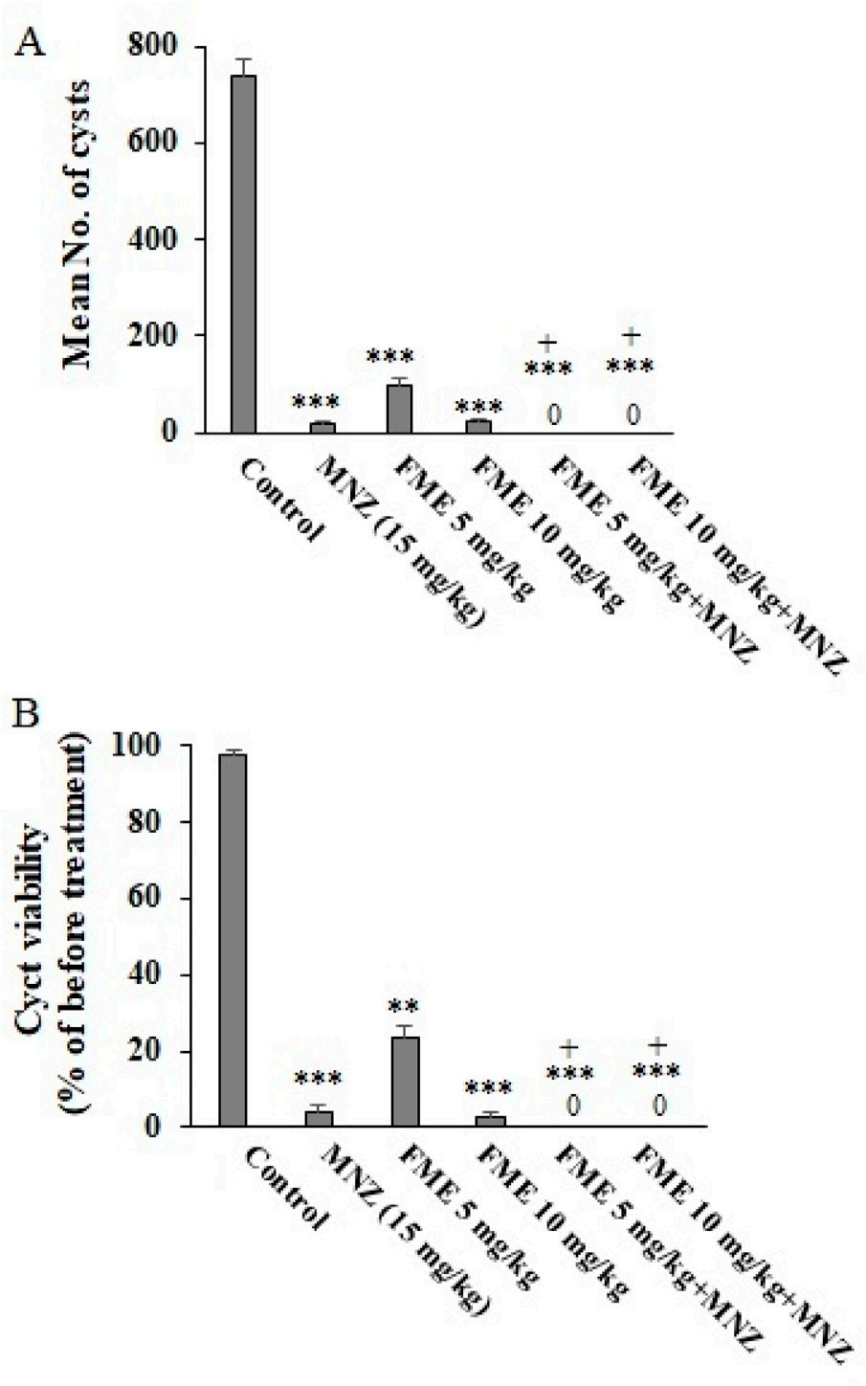
The *in vivo* effect of different doses of *Ferula macrecolea* essential oil (FME) on the number **(A)** and viability **(B)** of *Giardia lamblia* cysts in mice with giardiasis in comparison to metronidazole (MNZ). The results are presented as mean ± standard deviation (SD) with a sample size of n = 10. **P < 0.01; ***P < 0.001 compared to normal saline; + P < 0.05 compared to MNZ.

Following a 7-day administration of FME, the feces of infected mice were analyzed for the presence of *G. lamblia* cysts using the formalin-ether method. The percentage viability of cysts was assessed through eosin staining. As illustrated in Figure 2A, the application of various doses of FME, particularly in combination with MNZ (7.5 mg/kg/day) over the 7-day period, resulted in a statistically significant reduction (P < 0.001) in the average number of cysts. The observed reductions were 97.7%, 86.9%, 96.6%, 100%, and 100% in the groups treated with MNZ (15 mg/kg), FME 5 mg/kg, FME 10 mg/kg, FME 5 mg/kg + MNZ, FME 10 mg/kg + MNZ, respectively. Furthermore, Figure 2B demonstrates a significant decline (P < 0.001) in the viability of *Giardia* cysts following treatment with various doses of FME, particularly when combined with MNZ over the same 7-day period. The reductions in viability were recorded at 96.1%, 76.2%, 97.3%, 100% and 100% for the groups treated with MNZ (15 mg/kg), FME 5 mg/kg, FME 10 mg/kg, FME 5 mg/kg + MNZ, FME 10 mg/kg + MNZ, respectively.

### 3.5 Evaluating the serum level electrolytes

The biochemical analysis indicated a significant reduction in serum levels of Na and K in the infected mice (p < 0.05). Conversely, the oral administration of FME, particularly in combination with MNZ, resulted in a substantial modulation of serum Na and K levels in the infected mice (p < 0.001) when compared to the control group treated with normal saline (Table 3).

**TABLE 3:** *In vivo* effects of *Ferula macrecolea* essential oil (FME) on giardiasis in mice. Mean ± standard deviation (SD).

Drug	Sodium level (nmol/L)	Potassium level (nmol/L)
Normal saline	63.4 ± 5.6	3.4 ± 0.41
MNZ (15 mg/kg)	132.6 ± 7.6	7.3 ± 0.52
FME 5 mg/kg	102.3 ± 7.5	5.8 ± 0.36
FME 10 mg/kg	141.6 ± 6.9	6.8 ± 0.62
FME 5 mg/kg + MNZ	153.2 ± 8.3	7.4 ± 0.46
FME 10 mg/kg + MNZ	159.6 ± 7.9	7.9 ± 0.56

**P < 0.01; ***P < 0.001 compared to normal saline; + P < 0.05 compared to metronidazole (MNZ).

### 3.6 Evaluating the expression level of proinflammatory cytokines

**FIGURE 3:**
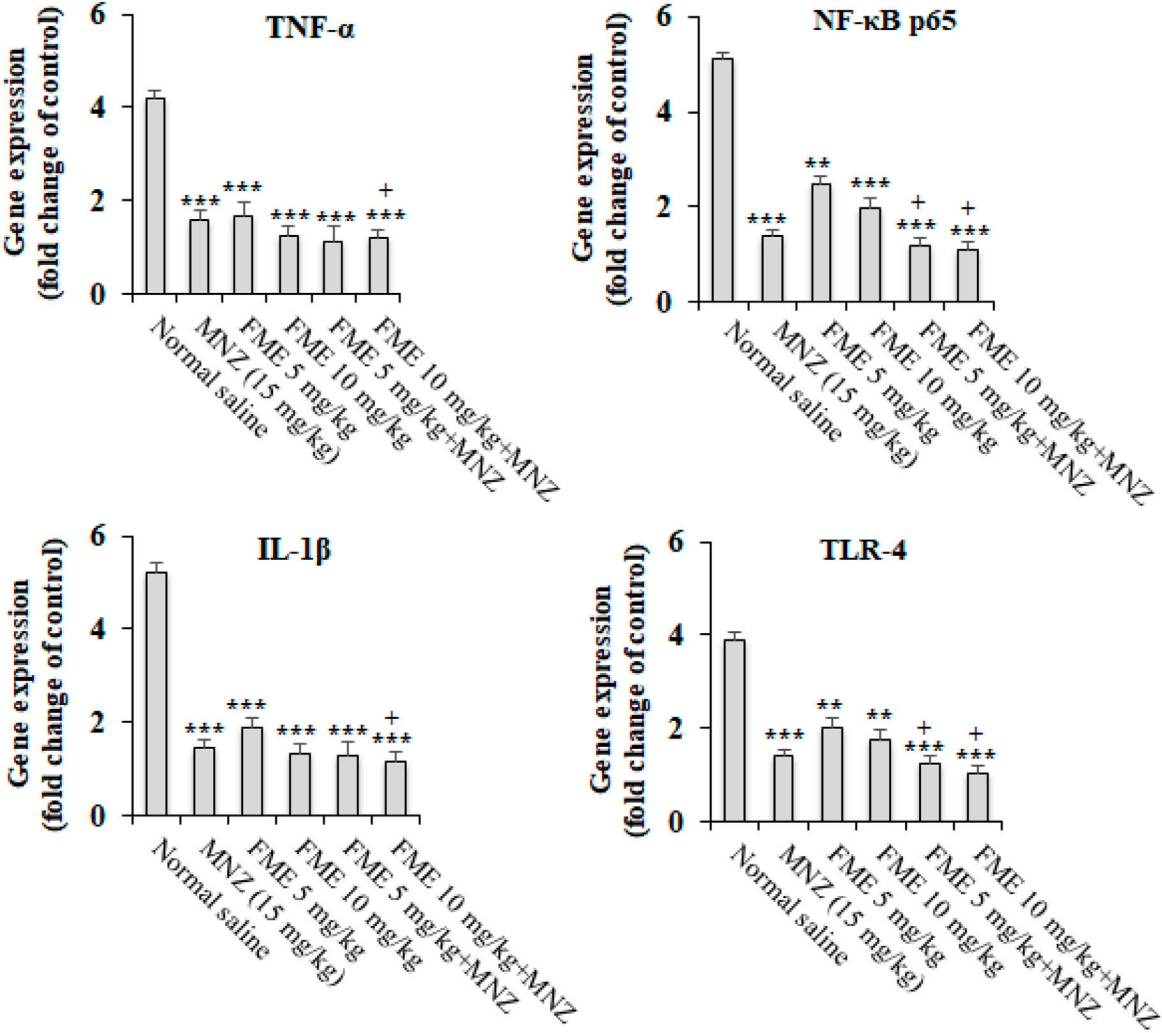
The *in vivo* effect of different doses of *Ferula macrecolea* essential oil (FME) on the expression level of interleukin-1 (IL-1), tumor necrosis factor-alpha (TNF-α), nuclear factor κB p65 (NF-κB p65), and Toll-like receptor 4 (TLR-4) in mice with giardiasis in comparison to metronidazole (MNZ). The results are presented as mean ± standard deviation (SD) with a sample size of n = 10. **P < 0.01; ***P < 0.001 compared to normal saline; + P < 0.05 compared to MNZ.

The findings from the real-time PCR analysis indicated that in the infected animal subjects, there was a notable elevation in the expression levels of the genes *IL-1β, TNF-α, NF-κB p65, and TLR-4* (p < 0.05). On the other hand, the administration of FME at dosages of 5 and 10 mg/kg, especially in combination with MNZ, resulted in a statistically significant decrease (P < 0.001) in the expression of the *IL-1β, TNF-α, NF-κB p65, and TLR-4* (Figure 3) in comparison to the infected mice that were treated with normal saline (P < 0.05).

## 4 Discussion

Today, the pharmacological management of giardiasis involves the administration of chemical agents, notably MNZ. Nonetheless, these agents are associated with a range of adverse effects, and their efficacy remains inconclusive such as carcinogenic and mutagenic properties, particularly affecting susceptible groups such as women and children, and are contraindicated for use during pregnancy (Watkins and Eckmann, 2014; Vivancos et al., 2018). The utilization of herbal medicines for addressing a range of health issues is experiencing significant growth globally. In particular, herbal medicinal products serve as the primary source of healthcare for a substantial portion of the population residing in developing nations (Alnomasy et al., 2021).

Our *in vitro* results showed that FME significantly diminished the viability and growth rate of *G. lamblia* trophozoites and cysts in a dose-dependent manner when compared to the control group. In addition, *in vivo* assay showed that the application of various doses of FME, particularly in combination with MNZ over the 7-day period, resulted in a statistically significant reduction in the mean number and viability of *Giardia* cysts. Numerous studies have demonstrated the antimicrobial properties of *Ferula* species against a diverse array of pathogenic bacteria (e.g., *Staphylococcus*., *Salmonella*) as well as fungi such as *Candida* and *Trichophyton* species (Boghrati and Iranshahi, 2019; Asili et al., 2009). In the term of antiparasitic activities, in a study by Mahmoudvand et al. (2022), the findings exhibited that FME had potent antileishmanial effects against promastigote and amastigotes of *Leishmania tropica* with IC_50_ of 27.6 and 42.3 μg/mL, respectively (Mahmoudvand et al., 2022). Another study conducted by Iranshahi et al. (2007) exhibited that *F. szowitsiana* acetone extract demonstrated considerable antileishmanial activity against *L. major* promastigotes, with an IC_50_ value of 11.8 μg/mL (Iranshahi et al., 2007). Another study conducted by Alyousif et al. (2021) revealed that FME demonstrated effective *in vitro* and *ex vivo* anthelminthic effects against *Echinococcus granulosus* protoscoleces, whereas entirely eliminating the parasites at concentrations of 150 and 300 μL/mL (Alyousif et al., 2021). In addition, Esmaeili et al. (2009) showed the strong antiparasitic properties of the *F. oopoda* extract against *Plasmodium falciparum* strains K1 and 3D7, exhibiting IC_50_ values of 26.6 μg/mL and 24.9 μg/mL, respectively (Esmaeili et al., 2009). Furthermore, Khanmohammadi et al. (2014) found that *F. szowitsiana* extract exhibited effective antiparasitic effects against *Trichomonas vaginalis* trophozoites, with an IC_50_ value of 0.360 mg/mL (Khanmohammadi et al., 2014). The discrepancies noted in the results can be ascribed to a variety of factors, including the specific type of parasite involved, the particular species of *Ferula* utilized, the characteristics of the extract employed, the concentration levels applied, and the methodologies implemented in the research studies.

The results of our GC/MS analysis indicate that the predominant compounds identified were terpinolene, n-nonanal, and linalool, respectively. Prior researches have established that the primary constituents of the essential oil derived from *Ferula* spp. are terpenoid compounds, including terpinolene, α-terpineol, α-pinene, β-pinene, myrcene, among others (Sahebkar and Iranshahi, 2011). Furthermore, existing literature suggests that the chemical composition of essential oils is influenced by various factors, including the geographical location and timing of plant collection, as well as the techniques employed in the extraction of the oils (Delfani et al., 2017). Terpenes and terpenoids are classified as hydrocarbon compounds that exhibit a range of pharmacological and therapeutic properties, particularly demonstrating antimicrobial activity against a diverse array of bacterial, fungal, viral, and parasitic strains (Guimarães et al., 2019; Mahizan et al., 2019). According to existing researches, terpene and terpenoid compounds demonstrate antimicrobial properties through various mechanisms of action, including the disruption of the cell wall, interference with oxygen consumption, and inhibition of virulence factors, among others (Guimarães et al., 2019; Mahizan et al., 2019; Srivastava and Singh, 2019).

Previous researches revealed that *Giardia* infection leads to malabsorption of glucose, sodium, and water, as well as a decrease in disaccharidase activity, which may be associated with a reduction in the absorptive surface area of epithelial cells (Buret, 2007). Our biochemical analyses indicated that the oral administration of FME, particularly in combination with MNZ, resulted in a substantial modulation of serum Na and K levels in the infected mice (p < 0.001) when compared to the control group treated with normal saline. These findings suggest that FME may have the potential to alleviate symptoms of giardiasis by regulating serum electrolyte levels in infected mice.

Researches have shown that *G. lamblia* elicits an inflammatory response marked by the secretion of IL-1β and TNF-α via various signaling pathways, including NF-κB p65, p38, and extracellular signal-regulated kinase (ERK) pathways (Pu et al., 2021). Prior investigations have established that the equilibrium and variations in cytokine concentrations can significantly affect or reflect clinical outcomes (Masoori et al., 2024). It has been proven that essential oils and their primary constituents modulate inflammatory responses through some pathways such as TLR pathways, as well as associated mitogen-activated protein kinase (MAPK) and ERK signaling pathways, and peroxisome proliferator-activated receptor gamma (PPAR-γ) signaling (Stojanović et al., 2024). We found that the administration of FME at dosages of 5 and 10 mg/kg, especially in combination with MNZ, resulted in a statistically significant decrease (P < 0.001) in the expression of the *L-1β, TNF-α, NF-κB p65, and TLR-4*. These findings suggest that these agents can effectively manage giardiasis in mice through their anti-inflammatory properties and suppressing specific inflammatory cytokines and NF-κB p65/TLR pathways.

In the context of cytotoxicity, the findings indicate that the FME significantly decreased cell viability in a dose-dependent manner for both cell types. While, the calculated CC_50_ values for FME against normal intestinal cells was determined to be 207.4 μg/mL. Similarly, a prior investigation revealed that the CC_50_ values for FME was 471.3 μg/mL against J774-A1 macrophage cells. They also reported that SI exceeding 10 for FME indicates a preferential efficacy against *L. tropica* amastigotes, while demonstrating limited cytotoxicity towards macrophage cells (Mahmoudvand et al., 2022).

## 5 Conclusion

This study’s results demonstrate the extract’s efficacy *in vitro* against *G. lamblia*, exhibiting minimal cytotoxicity towards normal cells. Furthermore, the extract was shown to manage giardiasis in murine models by modulating electrolyte levels and inflammatory responses via suppressing the NF-κB p65/TLR pathways. However, further research is necessary to clarify the specific efficacy and mechanisms of action of the extract in combating *G. lamblia* infection.

## Data Availability

The original contributions presented in the study are included in the article/supplementary material, further inquiries can be directed to the corresponding author.
